# Supplementary material for: Causal Relationship between Aging and Anorexia Nervosa: A White-Matter-Microstructure-Mediated Mendelian Randomization Analysis
Source: Biomedicines. 2024 Aug 16;12(8):1874. doi: 10.3390/biomedicines12081874 (PMC11351342; doi:10.3390/biomedicines12081874)
Supplement: Supplementary file 1 [file biomedicines-12-01874-s001.zip › Supplementary Figure/Figure S4/Singe SNP plot of MR analysis for TL to ubm-b-1568.pdf]

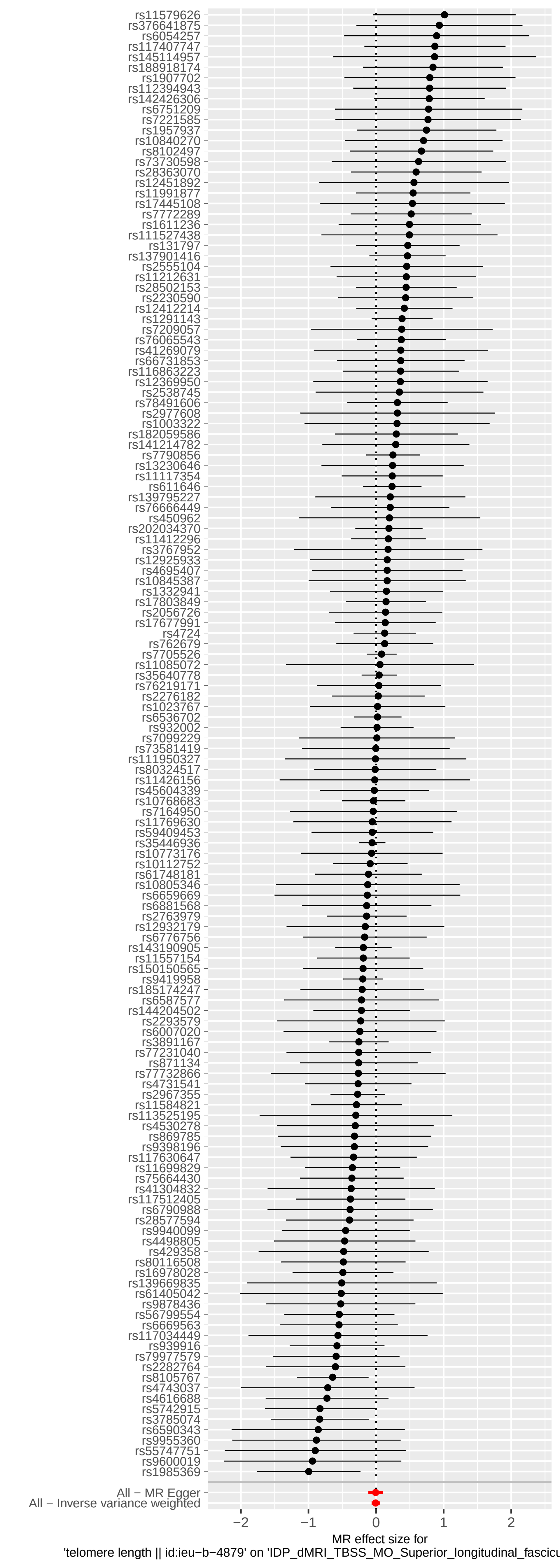
rs11579626
rs376641875
rs6054257
rs117407747
rs145114957
rs188918174
rs1907702
rs112394943
rs142426306
rs6751209
rs7221585
rs1957937
rs10840270
rs8102497
rs73730598
rs28363070
rs12451892
rs11991877
rs17445108
rs7772289
rs1611236
rs111527438
rs131797
rs137901416
rs2555104
rs11212631
rs28502153
rs2230590
rs12412214
rs1291143
rs7209057
rs76065543
rs41269079
rs66731853
rs116863223
rs12369950
rs2538745
rs78491606
rs2977608
rs1003322
rs182059586
rs141214782
rs7790856
rs13230646
rs11117354
rs611646
rs139795227
rs76666449
rs450962
rs202034370
rs11412296
rs3767952
rs12925933
rs4695407
rs10845387
rs1332941
rs17803849
rs2056726
rs17677991
rs4724
rs762679
rs7705526
rs11085072
rs35640778
rs76219171
rs2276182
rs1023767
rs6536702
rs932002
rs7099229
rs73581419
rs111950327
rs80324517
rs11426156
rs45604339
rs10768683
rs7164950
rs11769630
rs59409453
rs35446936
rs10773176
rs10112752
rs61748181
rs10805346
rs6659669
rs6881568
rs2763979
rs12932179
rs6776756
rs143190905
rs11557154
rs150150565
rs9419958
rs185174247
rs6587577
rs144204502
rs2293579
rs6007020
rs3891167
rs77231040
rs871134
rs77732866
rs4731541
rs2967355
rs11584821
rs113525195
rs4530278
rs869785
rs9398196
rs117630647
rs11699829
rs75664430
rs41304832
rs117512405
rs6790988
rs28577594
rs9940099
rs4498805
rs429358
rs80116508
rs16978028
rs139669835
rs61405042
rs9878436
rs56799554
rs6669563
rs117034449
rs939916
rs79977579
rs2282764
rs8105767
rs4743037
rs4616688
rs5742915
rs3785074
rs6590343
rs9955360
rs55747751
rs9600019
rs1985369
All − MR Egger
All − Inverse variance weighted
−2
−1
0
1
2
MR effect size for
'telomere length || id:ieu−b−4879' on 'IDP_dMRI_TBSS_MO_Superior_longitudinal_fascicu
